# T1-mapping accurately detects acute myocardial edema: a comparison to T2-weighted cardiovascular magnetic resonance imaging

**DOI:** 10.1186/1532-429X-14-S1-P290

**Published:** 2012-02-01

**Authors:** Vanessa Ferreira, Stefan K Piechnik, Erica Dall'Armellina, Theodoros Karamitsos, Jane M Francis, Robin P Choudhury, Matthias G Friedrich, Matthew D Robson, Stefan Neubauer

**Affiliations:** 1 Cardiovascular Medicine, University of Oxford, Oxford, UK; 2 Cardiac Sciences, Stephenson Cardiovascular MR Centre, Libin Cardiovascular Institute of Alberta, University of Calgary, Calgary, AB, Canada; 3 Cardiology, Université de Montréal, Montréal, QC, Canada

## Summary

Non-contrast T1-mapping using the novel ShMOLLI (Shortened Modified Look-Locker Inversion Recovery) sequence detects acute myocardial edema with high diagnostic accuracy.

## Background

T2-weighted cardiovascular magnetic resonance (CMR) is commonly used to detect myocardial edema. T1-mapping is also sensitive to changes in free water content and is quantitative, obviating the need for a presumed normal reference region to detect changes within affected myocardium. We hypothesized that T1-mapping using the novel sequence Shortened Modified Look-Locker Inversion Recovery (ShMOLLI) would have a higher diagnostic performance in detecting acute myocardial edema than dark-blood (STIR) and bright-blood (ACUT2E) T2-weighted CMR.

## Methods

We investigated 18 healthy controls (age 53 ± 14 years) and 19 patients (age 61 ± 10 years) presenting with acute myocardial stunning without infarction. Stunning was defined as acute cardiac symptoms associated with positive biomarkers for injury and acute wall motion abnormalities, but without late gadolinium enhancement (LGE). CMR performed within 9 days included cine, ShMOLLI T1-mapping, STIR, ACUT2E and LGE imaging. We analyzed wall motion, absolute T1 values and T2 signal intensity (SI) relative to both skeletal muscle (T2 SI myo:skeletal) and remote myocardium (T2 SI myo:remote).

## Results

**Figure 1:**
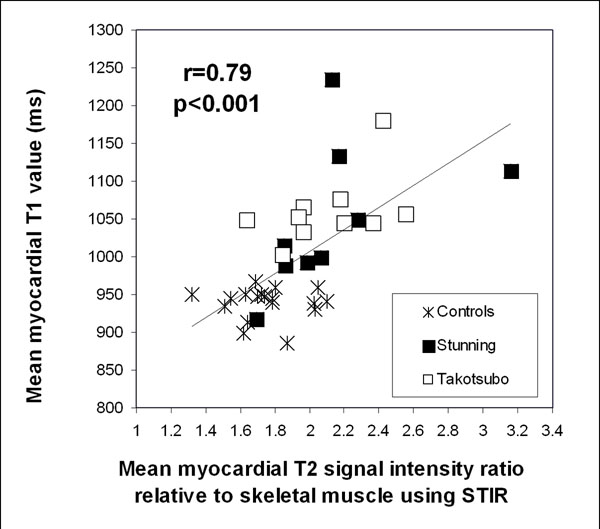
Correlation between mean myocardial T1 values and global myocardial T2 signal intensity (SI) relative to skeletal muscle ratio measured using STIR.

**Figure 2:**
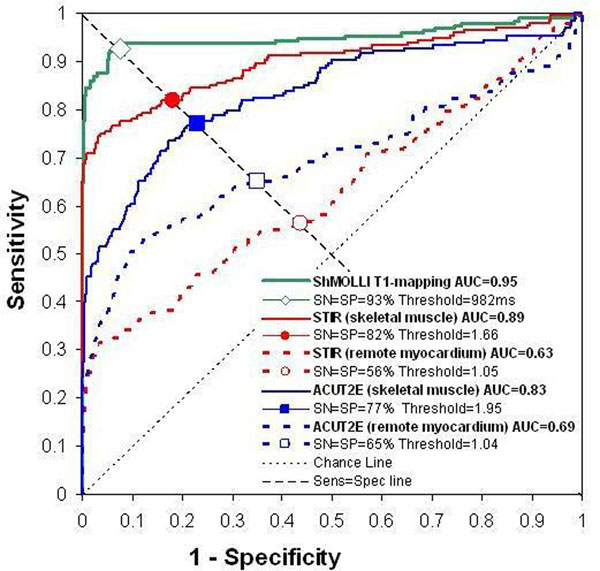
Receiver operator characteristic curves for the detection of acute myocardial edema by ShMOLLI T1-mapping, dark-blood STIR and bright-blood ACUT2E T2 imaging. For STIR and ACUT2E T2 imaging, reference regions of interest (skeletal muscle or remote myocardium) for comparison of myocardial signal intensity are in brackets. AUC=area under the curve; SP=specificity; SN=sensitivity.

Ten patients had Takotsubo cardiomyopathy and 9 had regional stunning as a final diagnosis by CMR. All had positive troponin levels (mean 6.92 ug/L, range 0.15 - 22.75 ug/L). No patients had significant LGE. There was good correlation between mean T1 values and mean T2 SI using skeletal muscle as a reference: r=0.79 for STIR (Figure 1) and r=0.63 for ACUT2E (both p<0.001). Receiver operating characteristics analysis was performed using patient segments with abnormal wall motion as surrogate “true positives” and control segments with normal wall motion as “true negatives” for myocardial edema (Figure 2), which showed that T1-mapping had a significantly larger area-under-the-curve (AUC=0.95) compared to STIR T2 SI myo:skeletal (0.89); ACUT2E T2 SI myo:skeletal (0.83); ACUT2E T2 SI myo:remote (0.69); and STIR T2 SI myo:remote (0.63). All comparisons were significant (p<0.03). Separately, in patients with regional stunning, where remote myocardium is used as the reference region for T2w-CMR, T1-mapping was as good as ACUT2E T2 SI myo:remote (AUC=0.92 vs. 0.96, p=0.24) which were superior to STIR T2 SI myo:remote (0.81, p<0.05).

## Conclusions

Non-contrast T1-mapping using ShMOLLI has a high diagnostic performance in detecting both focal and global myocardial edema compared to dark-blood and bright-blood T2-weighted CMR. T1-mapping may be used as a novel method to detect acute myocardial edema quantitatively, eliminating the need for subjective and presumed normal reference regions of interest.

## Funding

This study is funded by the Oxford National Institute for Health Research Biomedical Research Centre Programme. VMF is funded by the Alberta Heritage Foundation for Medical Research (AHFMR) and the University of Oxford Clarendon Fund Scholarship. Dr. Robin Choudhury is a Wellcome Trust Senior Research Fellow in Clinical Science. Stefan Neubauer and Robin Choudhury acknowledge support from the British Heart Foundation Centre of Research Excellence, Oxford.

